# Supplementary material for: Identification of a prefrontal cortex-to-amygdala pathway for chronic stress-induced anxiety
Source: Nat Commun. 2020 May 6;11:2221. doi: 10.1038/s41467-020-15920-7 (PMC7203160; doi:10.1038/s41467-020-15920-7)
Supplement: Supplementary file 1 — Supplementary Information [file 41467_2020_15920_MOESM1_ESM.pdf]

Supplementary Information

Identification of a prefrontal cortex-to-amygdala pathway for chronic stress-induced anxiety

Liu et al.

Supplementary Figure 1

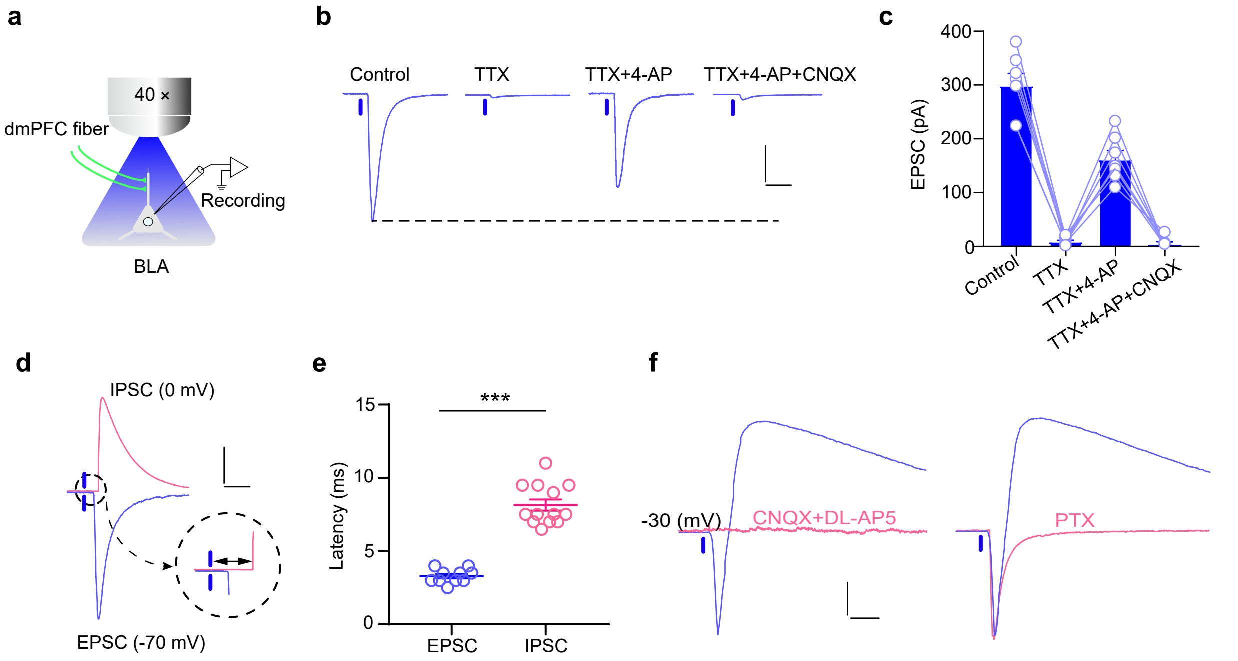

Supplementary Fig. 1 Synaptic identification of the evoked EPSCs and IPSCs in BLA PNs following light activation of dmPFC inputs. **a** Schematic showing light stimulation (LS) of dmPFC inputs to evoke synaptic response in BLA PNs. **b** Representative traces showing EPSCs when slices were sequentially perfused with ACSF (left), TTX (1μM, middle left), TTX + 4-AP (100 μM, middle right) and TTX + 4-AP + CNQX (20 μM, right). Cells were held at -70 mV. Scale bar = 5 ms, 100 pA. **c** Summary plots of the EPSC amplitudes in (b). n = 7 neurons /3 mice. **d** Representative traces of LS-evoked EPSCs and IPSCs in BLA PNs at -70 and 0 mV, respectively. Scale bar = 5 ms, 100 pA. **e** Summary plots of the synaptic latency of EPSCs and IPSCs. EPSC: n = 10 neurons /3 mice; IPSC: n = 13 neurons /5 mice. **f** Representative traces showing effects of CNQX (20 μM) and DL- AP5 (50 μM) or picrotoxin (100 μM) on LS-evoked EPSCs/IPSCs. Cells were held at -30 mV. Scale bar = 50 ms, 200 pA. Data are presented as mean ± SEM. Statistics are shown in Supplementary Table 1. ***p<0.001. Source data are provided as a Source Data file.

Supplementary Figure 2

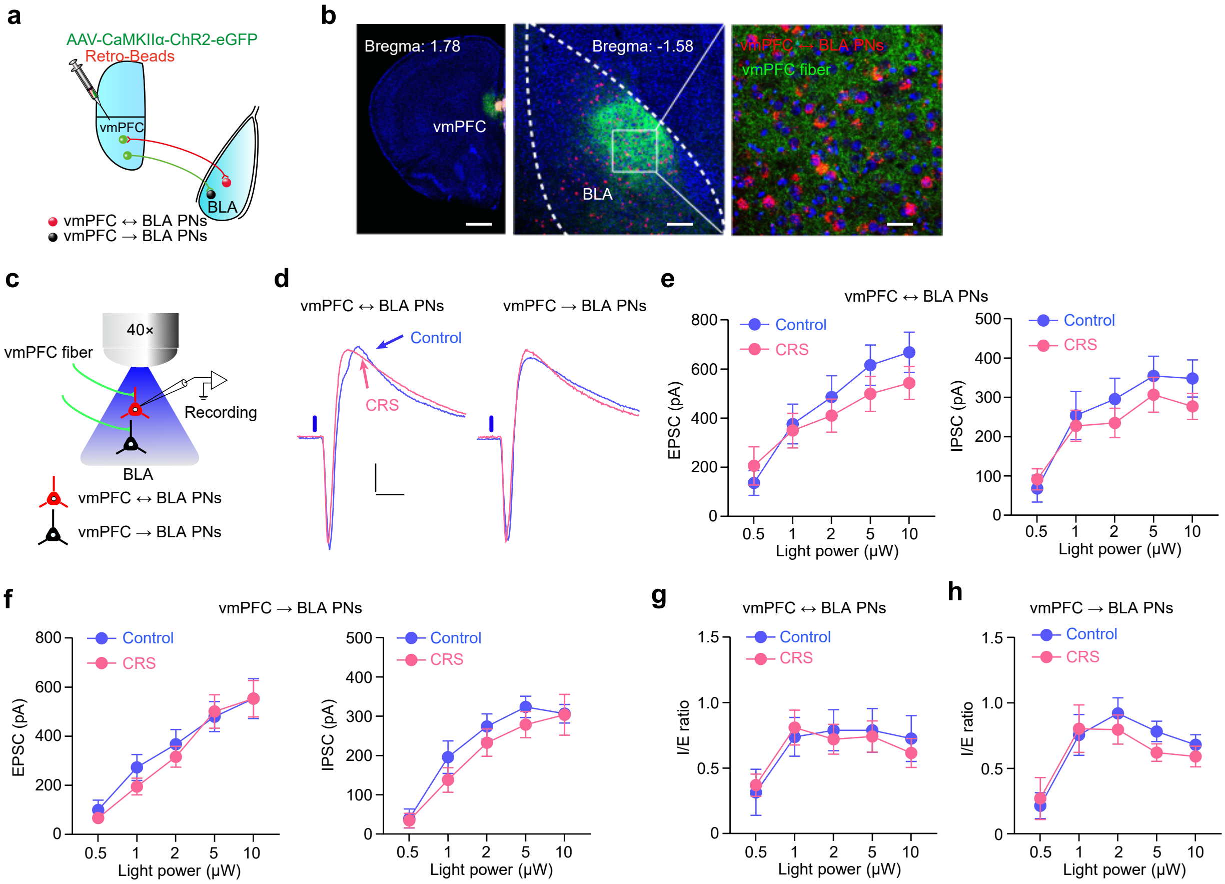

Supplementary Fig. 2 Chronic stress does not affect vmPFC-to-BLA transmission. **a** Schematic showing co-injection of ChR2-carrying AAV vectors and red fluorescent Retrobeads into vmPFC. Retrobeads were used to differentiate the putative vmPFC→BLA and vmPFC↔BLA PNs in BLA. **b** Representative images showing the injection site in vmPFC (left), and red Retrobeads-labeled vmPFC↔BLA PNs and vmPFC inputs in BLA (middle). The expanded square was shown on right. Scale bar: 500, 100 and 20 μm (left to right). **c** Schematic showing recording of the responses of vmPFC→BLA or vmPFC↔BLA PNs to optogenetic activation of vmPFC inputs. **d** Representative traces showing eEPSCs/eIPSCs in vmPFC↔BLA and vmPFC→BLA PNs following light activation of vmPFC inputs. Cells were held at -30 mV. Scale bar = 50 ms, 200 pA. **e** Summary plots of the light-evoked EPSCs (left) and IPSCs (right) in vmPFC↔BLA PNs with increasing light intensity. Control, n= 14 neurons/ 5 mice; CRS, n= 14 neurons/ 5 mice. **f** Same as in (e) except that the data were from vmPFC→BLA PNs. Control, n = 18 neurons/6 mice; CRS, n = 15 neurons/5 mice. **g** Summary plots of the IPSCs/EPSCs (I/E) ratio in vmPFC↔BLA PNs. Same sample size as in (e). **h** Summary plots of the I/E ratio in vmPFC→BLA PNs. Same sample size as in (f). Data are presented as mean ± SEM. Statistics are shown in Supplementary Table 1. Source data are provided as a Source Data file.

Supplementary Figure 3

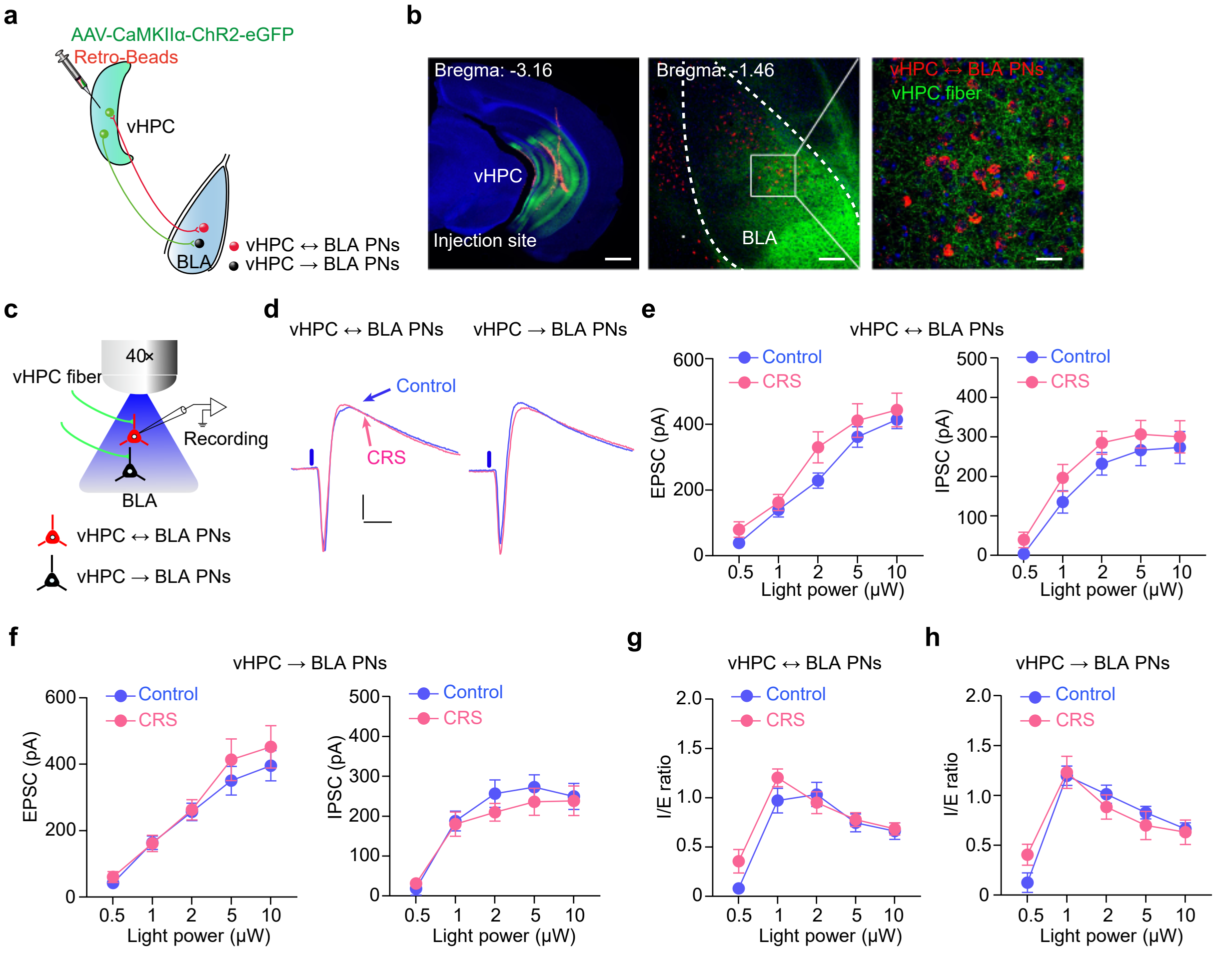

Supplementary Fig. 3 Chronic stress does not affect vHPC-to-BLA transmission. **a** Schematic showing co-injection of ChR2-carrying AAV vectors and red fluorescent Retrobeads into vHPC. Retrobeads were used to differentiate the putative vHPC→BLA and vHPC↔BLA PNs in BLA. **b** Representative images showing the injection sites in the vHPC (left) and red Retrobeads-labeled vHPC↔BLA PNs and vHPC inputs in BLA (middle). The expanded square was shown on right. Scale bar: 500, 100 and 20 μm (left to right). **c** Schematic showing recording of the responses of vHPC→BLA or vHPC↔BLA PNs to optogenetic activation of vHPC inputs. **d** Representative traces showing eEPSCs/eIPSCs in vHPC↔BLA and vHPC→BLA PNs following light activation of vHPC inputs. Cells were held at -30 mV. Scale bar = 50 ms, 150 pA. **e** Summary plots of the light-evoked EPSCs (left) and IPSCs (right) in vHPC↔BLA PNs with increasing light intensity. Control, n= 10 neurons/ 4 mice; CRS, n= 10 neurons/ 4 mice. **f** Same as in (e) except that the data were from vHPC→BLA PNs. Control, n = 11 neurons/4 mice; CRS, n = 10 neurons/4 mice. **g** Summary plots of the IPSCs/EPSCs (I/E) ratio in vHPC↔BLA PNs. Same sample size

as in (e). **h** Summary plots of the I/E ratio in vHPC→BLA PNs. Same sample size as in (f). Data are presented as mean ± SEM. Statistics are shown in Supplementary Table 1. Source data are provided as a Source Data file.

Supplementary Figure 4

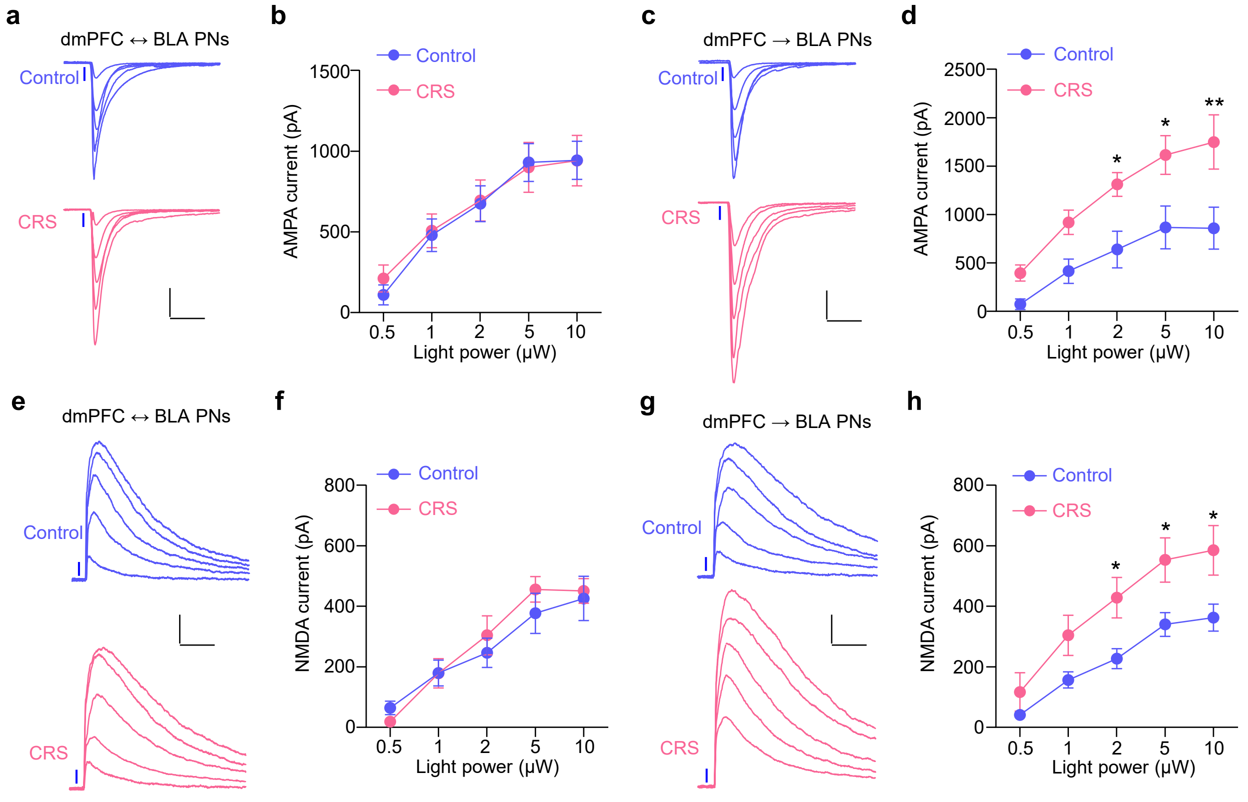

Supplementary Fig. 4 Chronic stress augments dmPFC-evoked glutamatergic transmission onto dmPFC→BLA PNs. **a** Representative traces showing light-evoked AMPA receptor currents with increasing light intensity in dmPFC↔BLA PNs. Cells were held at -70 mV in the presence of CGP52432 (5 µM) and picrotoxin (100 µM) to block GABAergic transmission. Scale bar = 10 ms, 300 pA. **b** Summary plots of the light-evoked AMPA receptor currents in dmPFC↔BLA PNs. Control, n = 13 neurons/5 mice; CRS, n = 12 neurons/4 mice. **c** Same as in (a) except that the data were from dmPFC→BLA PNs. Scale bar = 10 ms, 300 pA. **d** Summary plots of the light-evoked AMPA receptor currents in dmPFC→BLA PNs. Control, n = 12 neurons/4 mice; CRS, n = 11 neurons/4 mice. **e** Representative traces showing light-evoked NMDA receptor currents with increasing light intensity in dmPFC↔BLA PNs at +40 mV. Scale bar = 10 ms, 150 pA. **f** Summary plots of light-evoked NMDA receptor currents in dmPFC↔BLA PNs. Control, n =14 neurons/4 mice; CRS, n = 12 neurons/4 mice. **g** Same as in (e) except that the data were from dmPFC→BLA PNs. Scale bar = 10 ms, 150 pA. **h** Summary plots of light-evoked NMDA receptor currents in dmPFC→BLA PNs. Control, n = 14 neurons/5 mice; CRS, n = 13 neurons/4 mice. Data are presented as mean ± SEM. Statistics are shown in Supplementary Table 1. *p<0.05; **p<0.01. Source data are provided as a Source Data file.

Supplementary Figure 5

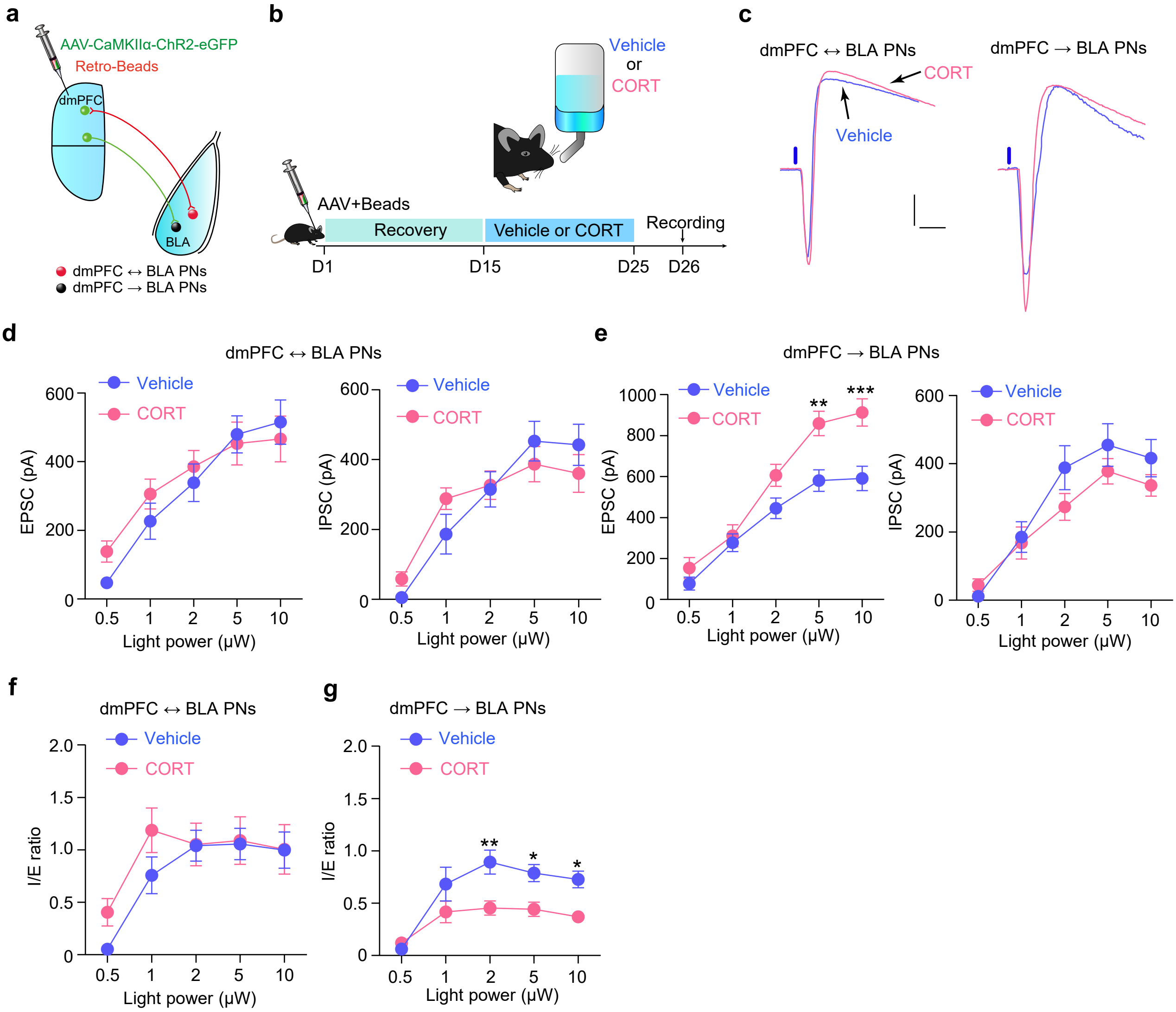

Supplementary Fig. 5 Chronically feeding the mice with corticosterone (CORT)-containing drinking water recapitulates the effects of chronic stress on dmPFC-to-BLA transmission. **a** Schematic showing the injection of red Retrobeads and ChR2-carrying AAV vector into dmPFC. **b** Schematic showing the experimental procedures for vehicle mice and mice subject to chronic CORT treatment. **c** Representative traces showing light-evoked EPSCs/IPSCs in dmPFC↔BLA and dmPFC→BLA PNs at -30 mV. Scale bar = 50 ms, 200 pA. **d** Summary plots of the light-evoked EPSCs (left) and IPSCs (right) in dmPFC↔BLA PNs. Vehicle, n = 10 neurons/4 mice; CORT, n = 13 neurons/4 mice. **e** Same as in (d) except that the data were from dmPFC→BLA PNs. Vehicle, n = 11 neurons/4 mice; CORT, n = 13 neurons/4 mice. **f** Summary plots of the IPSCs/EPSCs (I/E) ratio in dmPFC↔BLA PNs. Same sample size as in (d). **g** Summary plots of the I/E ratio in dmPFC→BLA PNs. Same sample size as in (e). Data are presented as mean ± SEM. Statistics are shown in Supplementary Table 1. *p<0.05; **p<0.01; ***p<0.001. Source data are provided as a Source Data file.

Supplementary Figure 6

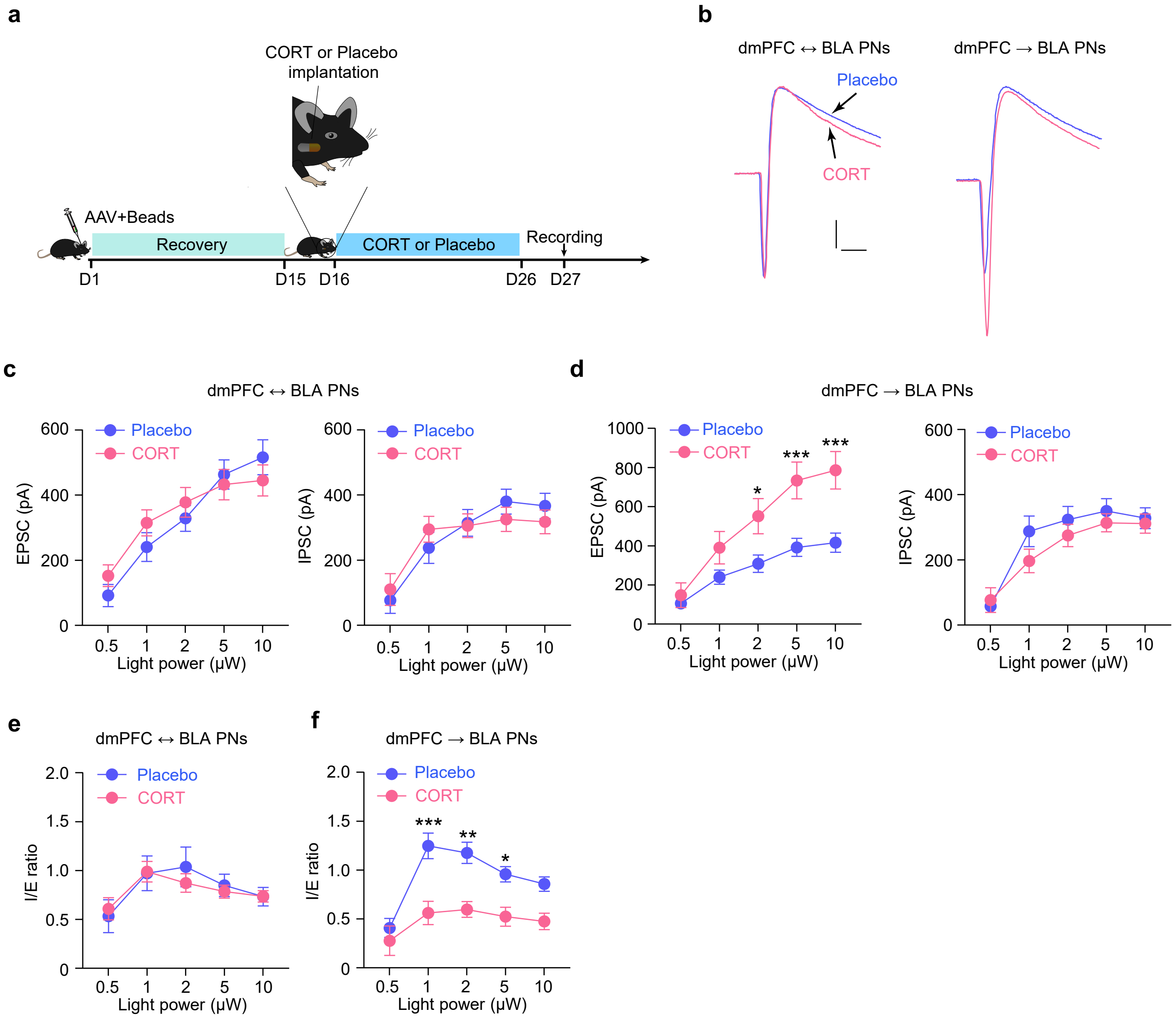

Supplementary Fig. 6 Chronic corticosterone (CORT) treatment with slow-release pellets recapitulates the effects of chronic stress on dmPFC-to-BLA transmission. **a** Schematic showing the experimental procedures for placebo mice and mice subject to chronic CORT treatment. **b** Representative traces showing light-evoked EPSCs/IPSCs in dmPFC↔BLA and dmPFC→BLA PNs at -30 mV. Scale bar = 50 ms, 150 pA. **c** Summary plots of the light-evoked EPSCs (left) and IPSCs (right) in dmPFC↔BLA PNs. Placebo, n = 11 neurons/4 mice; CORT, n = 12 neurons/4 mice. **d** Same as in (c) except that the data were from dmPFC→BLA PNs. Placebo, n = 16 neurons/5 mice; CORT, n = 12 neurons/4 mice. **e** Summary plots of the IPSCs/EPSCs (I/E) ratio in dmPFC↔BLA PNs. Same sample size as in (c). **f** Summary plots of the I/E ratio in dmPFC→BLA PNs. Same sample size as in (d). Data are presented as mean ± SEM. Statistics are shown in Supplementary Table 1. *p<0.05; **p<0.01; ***p<0.001. Source data are provided as a Source Data file.

Supplementary Figure 7

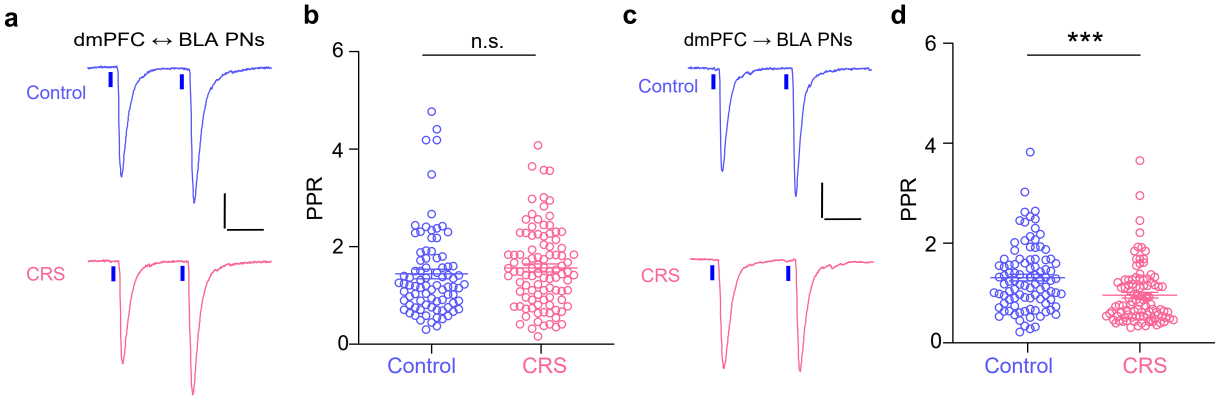

Supplementary Fig. 7 Recording from large cohort of BLA PNs reveals chronic stress increases prefrontal glutamate release onto BLA neurons that receive mono-directional dmPFC inputs (dmPFC→BLA PNs). **a** Representative traces showing EPSCs in dmPFC↔BLA PNs evoked by paired light stimuli of dmPFC inputs (100 ms interval). Scale bar = 50 ms, 50 pA. **b** Summary plots of PPR in dmPFC↔BLA PNs. Control, n = 85 neurons/23 mice; CRS, n = 99 neurons/27 mice. **c** Same as in (a) except that the data were from dmPFC→BLA PNs. Scale bar = 50 ms, 50 pA. **d** Summary plots of PPR in dmPFC→BLA PNs. Control, n = 96 neurons/23 mice; CRS, n = 99 neurons/27 mice. Data are presented as mean ± SEM. Statistics are shown in Supplementary Table 1. n.s., not significant; ***$p<0.001$. Source data are provided as a Source Data file.

Supplementary Figure 8

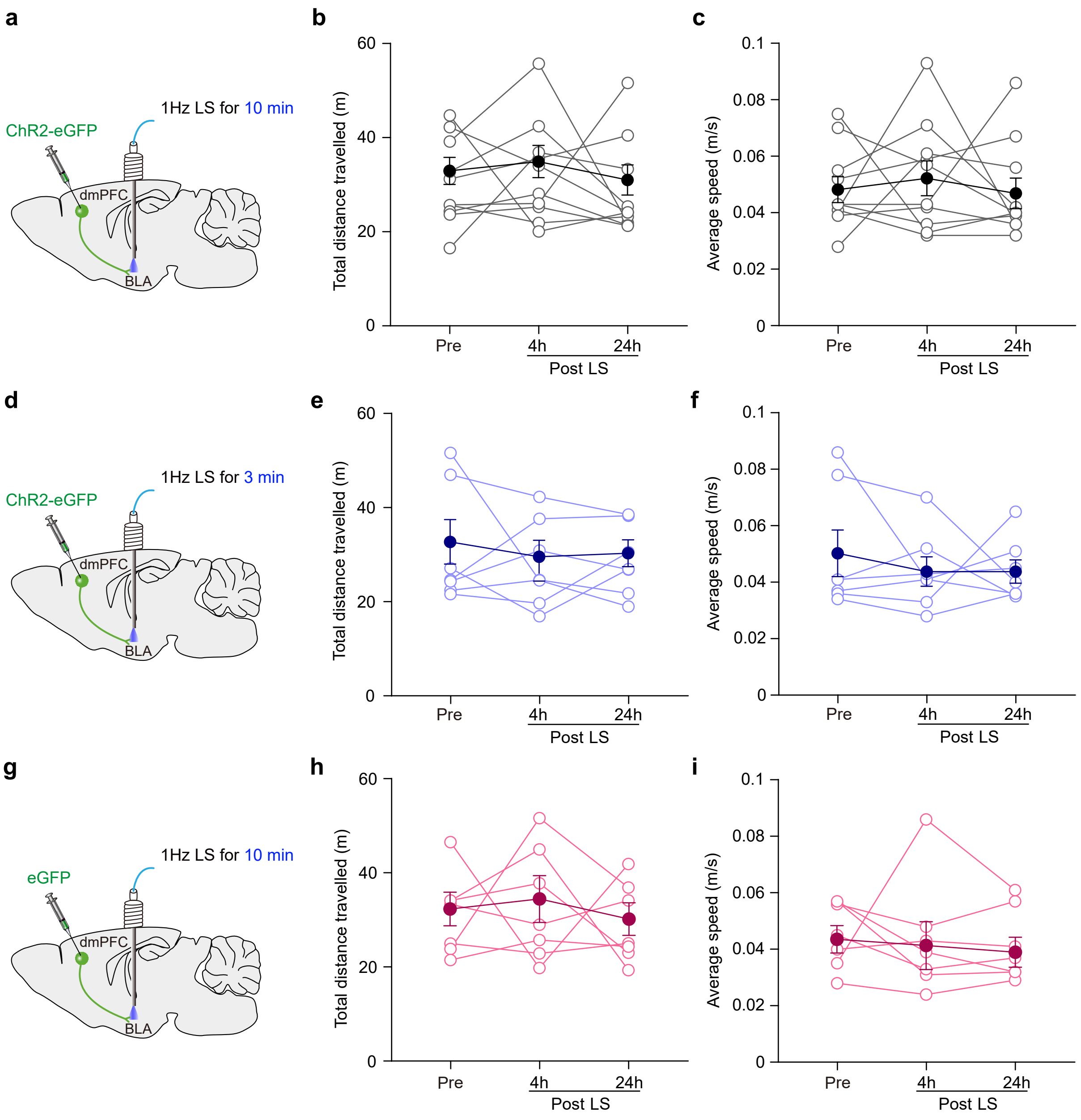

Supplementary Fig. 8 Activation of dmPFC-to-BLA pathway does not affect the total distance CRS mice travelled and their average speed. **a** Schematic illustration of injection of ChR2-carrying AAV in dmPFC and bilateral cannula implantation of optic fibers onto the BLA of CRS mice. 10 min LS were delivered. **b** Total distance measured pre- and post-LS. n = 10 mice. **c** Average speed measured pre- and post- LS. n = 10 mice. **d** Same as in (a) except that 3-min LS were delivered to BLA. **e** Total distance measured pre- and post-LS. n = 7 mice. **f** Average speed measured pre- and post-LS. n = 7 mice. **g** Same as in (a) except that the AAV vector carrying eGFP only was injected into the dmPFC of CRS mice. **h** Total distance measured pre- and post- LS. n = 7 mice. **i** Average speed measured pre- and post-LS. n = 7 mice. Data are presented as mean ± SEM. Statistics are shown in Supplementary Table 1. Source data are provided as a Source Data file.

Supplementary Figure 9

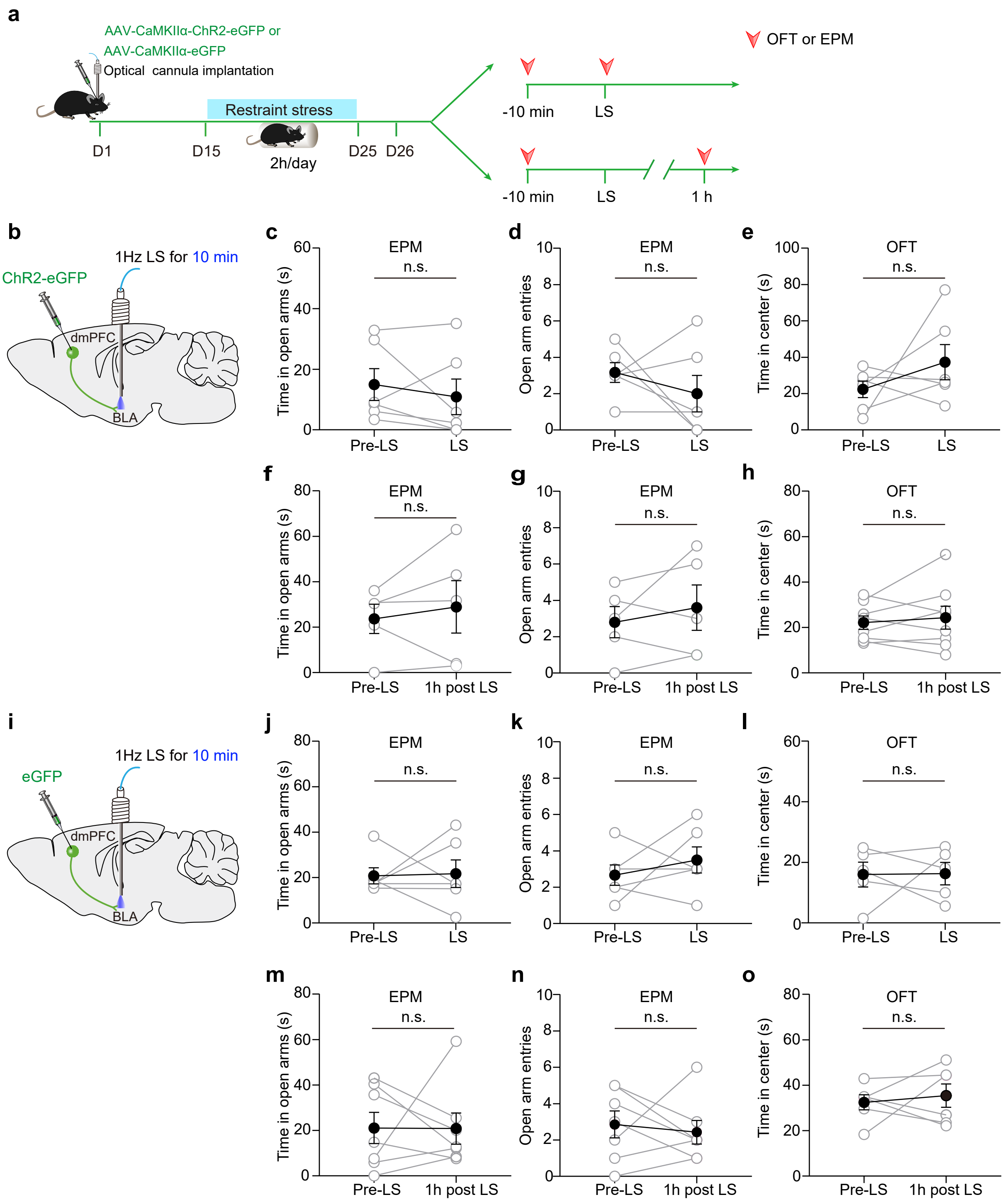

Supplementary Fig. 9 Activation of dmPFC-to-BLA pathway does not alter chronic stress-induced anxiety-like behavior as measured during light stimulation (LS) or 1h post LS. **a** Schematic showing the experimental procedures. **b** Schematic illustration of injection of ChR2-carrying AAV in dmPFC and bilateral cannula implantation with optic fibers onto BLA. LS (1 Hz, 10 min) was delivered to BLA. **c** EPM open arm time measured pre-LS and during LS. n = 6 mice. **d** EPM open arm entries measured pre-LS and during LS. n = 6 mice. **e** OFT time in center

measured pre-LS and during LS. n = 6 mice. **f** EPM open arm time measured pre-LS and 1h post LS . n = 5 mice. **g** EPM open arm entries measured pre-LS and 1h post LS. n = 5 mice. **h** OFT time in center measured pre-LS and 1h post LS. n = 8 mice. **i** Same as in (b) except that the AAV vectors containing eGFP only were injected into dmPFC. **j** EPM open arm time measured pre-LS and during LS. n = 6 mice. **k** EPM open arm entries measured pre-LS and during LS. n=6 mice. **l** OFT time in center measured pre-LS and during LS. n = 5 mice. **m** EPM open arm time measured pre-LS and 1h post LS. n = 7 mice. **n** EPM open arm entries measured pre-LS and 1h post LS. n = 7 mice. **o** OFT time in center measured pre-LS and 1h post LS. n = 6 mice. Data are presented as mean ± SEM. Statistics are shown in Supplementary Table 1. n.s., not significant. Source data are provided as a Source Data file.

Supplementary Table 1, Specific statistical tests used and the details of P-values for each experiment.

Figure 1	Sample size		Statistical Analysis	Treatment effect	P value	Significance
f	Control, n = 12 neurons/4 mice; CRS, n = 13 neurons /4 mice	Stress treatment	Two-way ANOVA with repeated measures	$F_{(1,23)} = 5.371$	P = 0.029	P < 0.05
		Light intensity	Two-way ANOVA with repeated measures	$F_{(4,92)} = 40.51$	P < 0.001	P < 0.001
		Interaction	Two-way ANOVA with repeated measures	$F_{(4,92)} = 4.495$	P = 0.002	P < 0.01
		Control vs CRS 0.5 μW	Bonferroni post hoc comparison		P > 0.999	n.s.
		Control vs CRS 1 μW	Bonferroni post hoc comparison		P = 0.865	n.s.
		Control vs CRS 2 μW	Bonferroni post hoc comparison		P = 0.114	n.s.
		Control vs CRS 5 μW	Bonferroni post hoc comparison		P = 0.019	P < 0.05
		Control vs CRS 10 μW	Bonferroni post hoc comparison		P = 0.012	P < 0.05
g	Same as in (f)	Stress treatment	Two-way ANOVA with repeated measures	$F_{(1,23)} = 0.217$	P = 0.645	n.s.
		Light intensity	Two-way ANOVA with repeated measures	$F_{(4,92)} = 42.12$	P < 0.001	P < 0.001
		Interaction	Two-way ANOVA with repeated measures	$F_{(4,92)} = 0.105$	P = 0.98	n.s.
h	Same as in (f)	Stress treatment	Two-way ANOVA with repeated measures	$F_{(1,23)} = 9.072$	P = 0.006	P < 0.01
		Light intensity	Two-way ANOVA with repeated measures	$F_{(4,92)} = 16.77$	P < 0.001	P < 0.001
		Interaction	Two-way ANOVA with repeated	$F_{(4,92)} = 0.995$	P = 0.414	n.s.

			measures			
		Control vs CRS 0.5 μW	Bonferroni post hoc comparison		P > 0.999	n.s.
		Control vs CRS 1 μW	Bonferroni post hoc comparison		P = 0.049	P < 0.05
		Control vs CRS 2 μW	Bonferroni post hoc comparison		P = 0.032	P < 0.05
		Control vs CRS 5 μW	Bonferroni post hoc comparison		P = 0.016	P < 0.05
		Control vs CRS 10 μW	Bonferroni post hoc comparison		P = 0.166	n.s.
m	Control, n = 12 neurons/4 mice; CRS, n = 14 neurons /5 mice	Stress treatment	Two-way ANOVA with repeated measures	$F_{(1,24)} = 0.0426$	P = 0.838	n.s.
		Light intensity	Two-way ANOVA with repeated measures	$F_{(4,96)} = 52.54$	P < 0.001	P < 0.001
		Interaction	Two-way ANOVA with repeated measures	$F_{(4,96)} = 0.837$	P = 0.505	n.s.
n	Same as in (m)	Stress treatment	Two-way ANOVA with repeated measures	$F_{(1,24)} = 0.457$	P = 0.506	n.s.
		Light intensity	Two-way ANOVA with repeated measures	$F_{(4,96)} = 47.67$	P < 0.001	P < 0.001
		Interaction	Two-way ANOVA with repeated measures	$F_{(4,96)} = 0.537$	P = 0.709	n.s.
o	Same as in (m)	Stress treatment	Two-way ANOVA with repeated measures	$F_{(1,24)} = 0.375$	P = 0.546	n.s.
		Light intensity	Two-way ANOVA with repeated measures	$F_{(4,96)} = 12.56$	P < 0.001	P < 0.001
		Interaction	Two-way ANOVA with repeated measures	$F_{(4,96)} = 1.27$	P = 0.287	n.s.
t	Control, n = 14 neurons/5	Stress treatment	Two-way ANOVA with repeated	$F_{(1,27)} = 0.077$	P = 0.780	n.s.

	mice; CRS, n = 15 neurons /5 mice		measures			
		Light intensity	Two-way ANOVA with repeated measures	$F_{(4,108)} = 70.56$	P < 0.001	P < 0.001
		Interaction	Two-way ANOVA with repeated measures	$F_{(4,108)} = 0.812$	P = 0.519	n.s.
u		Stress treatment	Two-way ANOVA with repeated measures	$F_{(1,27)} = 0.452$	P = 0.506	n.s.
		Light intensity	Two-way ANOVA with repeated measures	$F_{(4,108)} = 31.93$	P < 0.001	P < 0.001
		Interaction	Two-way ANOVA with repeated measures	$F_{(4,108)} = 1.136$	P = 0.343	n.s.
v		Stress treatment	Two-way ANOVA with repeated measures	$F_{(1,27)} = 0.009$	P = 0.924	n.s.
		Light intensity	Two-way ANOVA with repeated measures	$F_{(4,108)} = 19.8$	P < 0.001	P < 0.001
		Interaction	Two-way ANOVA with repeated measures	$F_{(4,108)} = 0.651$	P = 0.627	n.s.
**Figure 2**						
f: EPSC	Control, n = 14 neurons/4 mice; CRS, n = 15 neurons /4 mice	Stress treatment	Two-way ANOVA with repeated measures	$F_{(1,27)} = 0.354$	P = 0.557	n.s.
		Light intensity	Two-way ANOVA with repeated measures	$F_{(4,108)} = 58.45$	P < 0.001	P < 0.001
		Interaction	Two-way ANOVA with repeated measures	$F_{(4,108)} = 0.79$	P = 0.534	n.s.

f: IPSC	Control, n = 14 neurons/4 mice; CRS, n = 15 neurons /4 mice	Stress treatment	Two-way ANOVA with repeated measures	$F_{(1,27)} = 0.227$	P = 0.638	n.s.
		Light intensity	Two-way ANOVA with repeated measures	$F_{(4,108)} = 31.12$	P < 0.001	P < 0.001
		Interaction	Two-way ANOVA with repeated measures	$F_{(4,108)} = 0.979$	P = 0.422	n.s.
g: EPSC	Control, n =15 neurons/5 mice; CRS, n = 13 neurons /4 mice	Stress treatment	Two-way ANOVA with repeated measures	$F_{(1,26)} = 10.23$	P = 0.004	P < 0.01
		Light intensity	Two-way ANOVA with repeated measures	$F_{(4,104)} = 78.63$	P < 0.001	P < 0.001
		Interaction	Two-way ANOVA with repeated measures	$F_{(4,104)} = 14.09$	P < 0.001	P < 0.001
		Control vs CRS 0.5 μW	Bonferroni post hoc comparison		P > 0.999	n.s.
		Control vs CRS 1 μW	Bonferroni post hoc comparison		P > 0.999	n.s.
		Control vs CRS 2 μW	Bonferroni post hoc comparison		P = 0.037	P < 0.05
		Control vs CRS 5 μW	Bonferroni post hoc comparison		P < 0.001	P < 0.001
		Control vs CRS 10 μW	Bonferroni post hoc comparison		P < 0.001	P < 0.001
g: IPSC	Control, n =15 neurons/5 mice; CRS, n = 13 neurons /4 mice	Stress treatment	Two-way ANOVA with repeated measures	$F_{(1,26)} = 0.27$	P = 0.607	n.s.
		Light intensity	Two-way ANOVA with repeated measures	$F_{(4,104)} = 24.58$	P < 0.001	P < 0.001
		Interaction	Two-way ANOVA with repeated measures	$F_{(4,104)} = 0.318$	P = 0.865	n.s.

h	Same as in (f)	Stress treatment	Two-way ANOVA with repeated measures	$F_{(1,27)} = 0.245$	P = 0.625	n.s.
		Light intensity	Two-way ANOVA with repeated measures	$F_{(4,108)} = 7.153$	P < 0.001	P < 0.001
		Interaction	Two-way ANOVA with repeated measures	$F_{(4,108)} = 0.229$	P = 0.921	n.s.
i	Same as in (g)	Stress treatment	Two-way ANOVA with repeated measures	$F_{(1,26)} = 9.992$	P = 0.004	P < 0.01
		Light intensity	Two-way ANOVA with repeated measures	$F_{(4,104)} = 6.233$	P < 0.001	P < 0.001
		Interaction	Two-way ANOVA with repeated measures	$F_{(4,104)} = 0.971$	P = 0.427	n.s.
		Control vs CRS 0.5 μW	Bonferroni post hoc comparison		P = 0.579	n.s.
		Control vs CRS 1 μW	Bonferroni post hoc comparison		P = 0.015	P < 0.05
		Control vs CRS 2 μW	Bonferroni post hoc comparison		P = 0.023	P < 0.05
		Control vs CRS 5 μW	Bonferroni post hoc comparison		P = 0.016	P < 0.05
		Control vs CRS 10 μW	Bonferroni post hoc comparison		P = 0.039	P < 0.05
**Figure 3**						
b	Control, n = 13 neurons/5 mice; CRS, n = 14 neurons /5 mice	Stress treatment	Two-way ANOVA with repeated measures	$F_{(1,25)} = 0.351$	P = 0.559	n.s.
		Time interval	Two-way ANOVA with repeated measures	$F_{(3,75)} = 6.549$	P < 0.001	P < 0.001
		Interaction	Two-way ANOVA with repeated measures	$F_{(3,75)} = 0.332$	P = 0.802	n.s.
d	Control, n=14 neurons/5	Stress treatment	Two-way ANOVA with repeated	$F_{(1,25)} = 22.44$	P < 0.001	P < 0.001

	mice; CRS, n = 13neurons /5 mice		measures			
		Time interval	Two-way ANOVA with repeated measures	$F_{(3,75)} = 3.9$	P = 0.012	P < 0.05
		Interaction	Two-way ANOVA with repeated measures	$F_{(3,75)} = 0.14$	P = 0.936	n.s.
		Control vs CRS 50 ms	Bonferroni post hoc comparison		P = 0.005	P < 0.01
		Control vs CRS 100 ms	Bonferroni post hoc comparison		P = 0.029	P < 0.05
		Control vs CRS 200 ms	Bonferroni post hoc comparison		P = 0.011	P < 0.05
		Control vs CRS 500 ms	Bonferroni post hoc comparison		P = 0.045	P < 0.05
f	Control, n = 9 neurons/4 mice; CRS, n = 10 neurons /4 mice	Control vs CRS	Two-tailed unpaired t test		p = 0.535	n.s.
h	Control, n = 10 neurons/4 mice; CRS, n = 9 neurons/4 mice	Control vs CRS	Two-tailed unpaired t test		p = 0.016	P < 0.05
**Figure 4**						
**b**	Control, n = 14 neurons/5 mice; CRS, n = 11 neurons /4 mice	Control vs CRS	Two-tailed unpaired t test		p = 0.978	n.s.
d	Control, n = 16 neurons/6 mice; CRS, n = 13 neurons /4 mice	Control vs CRS	Two-tailed unpaired t test		p = 0.746	n.s.
f	Control, n = 14 neurons/4 mice; CRS, n = 12 neurons /4 mice	Stress treatment	Two-way ANOVA with repeated measures	$F_{(1,24)} = 0.03$	P = 0.862	n.s.

		Voltage	Two-way ANOVA with repeated measures	$F_{(6,144)} = 547$	$P < 0.001$	$P < 0.001$
		Interaction	Two-way ANOVA with repeated measures	$F_{(6,144)} = 0.972$	$P = 0.447$	n.s.
g	Same as in (f)	Stress treatment	Two-way ANOVA with repeated measures	$F_{(1,24)} = 0.09$	$P = 0.757$	n.s.
		Voltage	Two-way ANOVA with repeated measures	$F_{(6,144)} = 867.9$	$P < 0.001$	$P < 0.001$
		Interaction	Two-way ANOVA with repeated measures	$F_{(6,144)} = 1.874$	$P = 0.089$	n.s.
i	Control, n = 13 neurons/5 mice; CRS, n = 14 neurons /5 mice	Stress treatment	Two-way ANOVA with repeated measures	$F_{(1,25)} = 1.755$	$P = 0.197$	n.s.
		Voltage	Two-way ANOVA with repeated measures	$F_{(6,150)} = 1509$	$P < 0.001$	$P < 0.001$
		Interaction	Two-way ANOVA with repeated measures	$F_{(6,150)} = 0.566$	$P = 0.756$	n.s.
j	Same as in (i)	Stress treatment	Two-way ANOVA with repeated measures	$F_{(1,25)} = 1.91$	$P = 0.179$	n.s.
		Voltage	Two-way ANOVA with repeated measures	$F_{(6,150)} = 1049$	$P < 0.001$	$P < 0.001$
		Interaction	Two-way ANOVA with repeated measures	$F_{(6,150)} = 1.823$	$P = 0.090$	n.s.
l	Control, n= 15 neurons/5 mice; CRS, n = 13 neurons /4 mice	Control vs CRS	Two-tailed unpaired t test		$P = 0.919$	n.s.

m	Same as in (l)	Control vs CRS	Two-tailed unpaired t test		P = 0.200	n.s.
o	Control, n =17 neurons/6 mice; CRS, n = 15 neurons /5 mice	Control vs CRS	Two-tailed unpaired t test		P = 0.003	P < 0.01
p	Same as in (o)	Control vs CRS	Two-tailed unpaired t test		P = 0.411	n.s.
**Figure 5**						
d	Control, n = 11 mice; CRS, n = 13 mice	Control vs CRS	Two-tailed unpaired t test		P = 0.046	P < 0.05
e	Same as in (d)	Control vs CRS	Two-tailed unpaired t test		P = 0.042	P < 0.05
f	Control, n = 12 mice; CRS, n = 14 mice	Control vs CRS	Two-tailed unpaired t test		P = 0.007	P < 0.01
g	Same as in (f)	Control vs CRS	Two-tailed unpaired t test		P = 0.231	n.s.
**Figure 6**						
c	n = 9 neurons/6 mice	dmPFC↔BLA PNs	One-way ANOVA with repeated measures	$F_{(4,32)} = 1.143$	P = 0.354	n.s.
	n = 10 neurons/7 mice	dmPFC→BLA PNs	One-way ANOVA with repeated measures	$F_{(4,36)} = 0.772$	P = 0.550	n.s.
e	n= 9 neurons/5 mice	dmPFC↔BLA PNs	One-way ANOVA with repeated measures	$F_{(4,32)} = 2.646$	P = 0.052	n.s.
	n = 9 neurons/6 mice	dmPFC→BLA PNs	One-way ANOVA with repeated measures	$F_{(4,32)} = 5.032$	P = 0.003	P < 0.01
		Pre- vs Post-LS 1 min	Bonferroni post hoc comparison		P = 0.002	P < 0.01
		Pre- vs Post-LS 10 min	Bonferroni post hoc comparison		P = 0.049	P < 0.05
		Pre- vs Post-LS 20 min	Bonferroni post hoc comparison		P = 0.004	P < 0.01
		Pre- vs Post-LS 30 min	Bonferroni post hoc comparison		P = 0.009	P < 0.01
h	CRS, n = 14 neurons/4		One-way ANOVA	$F_{(2,38)} = 0.353$	P = 0.705	n.s.

	mice; CRS + LS 3 min, n = 14 neurons/5 mice; CRS + LS 10 min, n = 13 neurons /4 mice					
i	CRS, n = 16 neurons/5 mice; CRS + LS 3 min, n = 13 neurons/4 mice; CRS + LS 10 min, n = 16 neurons /5 mice		One-way ANOVA	$F_{(2,42)} = 6.91$	P = 0.003	P < 0.01
		CRS vs CRS + LS 3 min	Bonferroni post hoc comparison		P > 0.999	n.s.
		CRS vs CRS + LS 10 min	Bonferroni post hoc comparison		P = 0.006	P < 0.01
k	CRS, n = 15 neurons/5 mice; CRS + LS 3 min, n = 13 neurons/4 mice; CRS + LS 10 min, n = 13 neurons /4 mice		One-way ANOVA	$F_{(2,38)} = 0.461$	P = 0.634	n.s.
l	CRS, n=16 neurons/5 mice; CRS + LS 3 min, n =15 neurons/5 mice; CRS + LS 10 min, n = 14 neurons/4 mice		One-way ANOVA	$F_{(2,42)} = 1.044$	P = 0.361	n.s.
**Figure 7**						
d	n = 7 mice		One-way ANOVA with repeated measures	$F_{(2,12)} = 4.714$	P = 0.031	P < 0.05
		Pre- vs Post- LS 4 h	Bonferroni post hoc comparison		P = 0.019	P < 0.05
		Pre- vs Post- LS 24 h	Bonferroni post hoc comparison		P = 0.337	n.s.

e	n = 7 mice		One-way ANOVA with repeated measures	$F_{(2,12)} = 3.509$	p = 0.063	n.s.
		Pre- vs Post- LS 4 h	Bonferroni post hoc comparison		P = 0.060	n.s.
		Pre- vs Post- LS 24 h	Bonferroni post hoc comparison		P = 0.080	n.s.
f	n = 10 mice		One-way ANOVA with repeated measures	$F_{(2,18)} = 6.992$	P = 0.006	P < 0.01
		Pre- vs Post- LS 4 h	Bonferroni post hoc comparison		P = 0.0499	P < 0.05
		Pre- vs Post- LS 24 h	Bonferroni post hoc comparison		P = 0.004	P < 0.01
h	n = 6 mice		One-way ANOVA with repeated measures	$F_{(2,10)} = 0.793$	P = 0.478	n.s.
i	n = 6 mice		One-way ANOVA with repeated measures	$F_{(2,10)} = 0.195$	P = 0.826	n.s.
j	n = 7 mice		One-way ANOVA with repeated measures	$F_{(2,12)} = 0.465$	P = 0.638	n.s.
l	n = 6 mice		One-way ANOVA with repeated measures	$F_{(2,10)} = 0.872$	P = 0.447	n.s.
m	n = 6 mice		One-way ANOVA with repeated measures	$F_{(2,10)} = 2.067$	P = 0.177	n.s.
n	n = 7 mice		One-way ANOVA with repeated measures	$F_{(2,12)} = 0.45$	P = 0.648	n.s.
**Supplementary Figure 1**						
e	EPSC: n = 10 neurons/3 mice; IPSC: n = 13 neurons /5 mice	EPSC vs IPSC	Two-tailed unpaired t test		P < 0.001	P < 0.001
**Supplementary Figure 2**						
e: EPSC	Control, n = 14 neurons/5	Stress treatment	Two-way ANOVA with repeated	$F_{(1,26)} = 0.324$	P = 0.574	n.s.

	mice; CRS, n = 14 neurons /5 mice		measures			
		Light intensity	Two-way ANOVA with repeated measures	$F_{(4,104)}$ = 53.19	P < 0.001	P < 0.001
		Interaction	Two-way ANOVA with repeated measures	$F_{(4,104)}$ = 2.86	P = 0.027	P < 0.05
e: IPSC	Control, n = 14 neurons/5 mice; CRS, n = 14 neurons /5 mice	Stress treatment	Two-way ANOVA with repeated measures	$F_{(1,26)}$ = 0.541	P = 0.469	n.s.
		Light intensity	Two-way ANOVA with repeated measures	$F_{(4,104)}$ = 22.92	P < 0.001	P < 0.001
		Interaction	Two-way ANOVA with repeated measures	$F_{(4,104)}$ = 0.812	P = 0.521	n.s.
f: EPSC	Control, n = 18 neurons/6 mice; CRS, n = 15 neurons /5 mice	Stress treatment	Two-way ANOVA with repeated measures	$F_{(1,31)}$ = 0.175	P = 0.679	n.s.
		Light intensity	Two-way ANOVA with repeated measures	$F_{(4,124)}$ = 55.31	P < 0.001	P < 0.001
		Interaction	Two-way ANOVA with repeated measures	$F_{(4,124)}$ = 0.587	P = 0.673	n.s.
f: IPSC	Control, n = 18 neurons/6 mice; CRS, n = 15 neurons /5 mice	Stress treatment	Two-way ANOVA with repeated measures	$F_{(1,31)}$ = 0.78	P = 0.384	n.s.
		Light intensity	Two-way ANOVA with repeated measures	$F_{(4,124)}$ = 41.44	P < 0.001	P < 0.001
		Interaction	Two-way ANOVA with repeated	$F_{(4,124)}$ = 0.507	P = 0.730	n.s.

			measures			
g	Same as in (e)	Stress treatment	Two-way ANOVA with repeated measures	$F_{(1,26)}$ =0.02	P = 0.889	n.s.
		Light intensity	Two-way ANOVA with repeated measures	$F_{(4,104)}$ = 5.4	P < 0.001	P < 0.001
		Interaction	Two-way ANOVA with repeated measures	$F_{(4,104)}$ = 0.253	P = 0.907	n.s.
h	Same as in (f)	Stress treatment	Two-way ANOVA with repeated measures	$F_{(1,31)}$ = 0.205	P = 0.654	n.s.
		Light intensity	Two-way ANOVA with repeated measures	$F_{(4,124)}$ = 13.42	P < 0.001	P < 0.001
		Interaction	Two-way ANOVA with repeated measures	$F_{(4,124)}$ = 0.589	P = 0.671	n.s.
**Supplementary Figure 3**						
e: EPSC	Control, n = 10 neurons/4 mice; CRS, n = 10 neurons /4 mice	Stress treatment	Two-way ANOVA with repeated measures	$F_{(1,18)}$ =2.049	P = 0.169	n.s.
		Light intensity	Two-way ANOVA with repeated measures	$F_{(4,72)}$ = 67.56	P < 0.001	P < 0.001
		Interaction	Two-way ANOVA with repeated measures	$F_{(4,72)}$ = 0.676	P = 0.611	n.s.
e: IPSC	Control, n = 10 neurons/4 mice; CRS, n = 10 neurons /4 mice	Stress treatment	Two-way ANOVA with repeated measures	$F_{(1,18)}$ =1.761	P = 0.201	n.s.
		Light intensity	Two-way ANOVA with repeated measures	$F_{(4,72)}$ = 43.34	P < 0.001	P < 0.001

		Interaction	Two-way ANOVA with repeated measures	$F_{(4,72)} = 0.157$	$P = 0.959$	n.s.
f: EPSC	Control, n = 11 neurons/4 mice; CRS, n = 10 neurons /4 mice	Stress treatment	Two-way ANOVA with repeated measures	$F_{(1,19)} = 0.525$	$P = 0.478$	n.s.
		Light intensity	Two-way ANOVA with repeated measures	$F_{(4,76)} = 53.87$	$P < 0.001$	$P < 0.001$
		Interaction	Two-way ANOVA with repeated measures	$F_{(4,76)} = 0.505$	$P = 0.732$	n.s.
f: IPSC	Control, n = 11 neurons/4 mice; CRS, n = 10 neurons /4 mice	Stress treatment	Two-way ANOVA with repeated measures	$F_{(1,19)} = 0.382$	$P = 0.544$	n.s.
		Light intensity	Two-way ANOVA with repeated measures	$F_{(4,76)} = 35.75$	$P < 0.001$	$P < 0.001$
		Interaction	Two-way ANOVA with repeated measures	$F_{(4,76)} = 0.559$	$P = 0.693$	n.s.
g	Same as in (e)	Stress treatment	Two-way ANOVA with repeated measures	$F_{(1,18)} = 1.135$	$P = 0.301$	n.s.
		Light intensity	Two-way ANOVA with repeated measures	$F_{(4,72)} = 36.45$	$P < 0.001$	$P < 0.001$
		Interaction	Two-way ANOVA with repeated measures	$F_{(4,72)} = 1.834$	$P = 0.132$	n.s.
h	Same as in (f)	Stress treatment	Two-way ANOVA with repeated measures	$F_{(1,19)} = 0.002$	$P = 0.961$	n.s.
		Light intensity	Two-way ANOVA with repeated measures	$F_{(4,76)} = 28.47$	$P < 0.001$	$P < 0.001$

		Interaction	Two-way ANOVA with repeated measures	$F_{(4,76)} = 1.625$	P = 0.177	n.s.
**Supplementary Figure 4**						
b	Control, n = 13 neurons/5 mice; CRS, n = 12 neurons /4 mice	Stress treatment	Two-way ANOVA with repeated measures	$F_{(1,23)} = 0.025$	P = 0.875	n.s.
		Light intensity	Two-way ANOVA with repeated measures	$F_{(4,92)} = 64.5$	P < 0.001	P < 0.001
		Interaction	Two-way ANOVA with repeated measures	$F_{(4,92)} = 0.371$	P = 0.829	n.s.
d	Control, n = 12 neurons/4 mice; CRS, n = 11 neurons /4 mice	Stress treatment	Two-way ANOVA with repeated measures	$F_{(1,21)} = 10.83$	P = 0.003	P < 0.01
		Light intensity	Two-way ANOVA with repeated measures	$F_{(4,84)} = 25.29$	P < 0.001	P < 0.001
		Interaction	Two-way ANOVA with repeated measures	$F_{(4,84)} = 1.567$	P = 0.195	n.s.
		Control vs CRS 0.5 μW	Bonferroni post hoc comparison		P = 0.971	n.s.
		Control vs CRS 1 μW	Bonferroni post hoc comparison		P = 0.220	n.s.
		Control vs CRS 2 μW	Bonferroni post hoc comparison		P = 0.038	P < 0.05
		Control vs CRS 5 μW	Bonferroni post hoc comparison		P = 0.016	P < 0.05
		Control vs CRS 10 μW	Bonferroni post hoc comparison		P = 0.002	P < 0.01
f	Control, n = 14 neurons/4 mice; CRS, n = 12 neurons /4 mice	Stress treatment	Two-way ANOVA with repeated measures	$F_{(1,24)} = 0.16$	P = 0.693	n.s.
		Light intensity	Two-way ANOVA with repeated	$F_{(4,96)} = 50.94$	P < 0.001	P < 0.001

			measures			
		Interaction	Two-way ANOVA with repeated measures	$F_{(4,96)} = 1.117$	P = 0.353	n.s.
h	Control, n = 14 neurons/5 mice; CRS, n = 13 neurons /4 mice	Stress treatment	Two-way ANOVA with repeated measures	$F_{(1,25)} = 7.891$	P = 0.009	P < 0.01
		Light intensity	Two-way ANOVA with repeated measures	$F_{(4,100)} = 40.92$	P < 0.001	P < 0.001
		Interaction	Two-way ANOVA with repeated measures	$F_{(4,100)} = 1.451$	P = 0.223	n.s.
		Control vs CRS 0.5 μW	Bonferroni post hoc comparison		P > 0.999	n.s.
		Control vs CRS 1 μW	Bonferroni post hoc comparison		P = 0.275	n.s.
		Control vs CRS 2 μW	Bonferroni post hoc comparison		P = 0.047	P < 0.05
		Control vs CRS 5 μW	Bonferroni post hoc comparison		P = 0.030	P < 0.05
		Control vs CRS 10 μW	Bonferroni post hoc comparison		P = 0.021	P < 0.05
**Supplementary Figure 5**						
d:EPSC	Vehicle, n = 10 neurons/4 mice; CORT, n = 13 neurons /4 mice	CORT treatment	Two-way ANOVA with repeated measures	$F_{(1,21)} = 0.213$	P = 0.650	n.s.
		Light intensity	Two-way ANOVA with repeated measures	$F_{(4,84)} = 47.08$	P < 0.001	P < 0.001
		Interaction	Two-way ANOVA with repeated measures	$F_{(4,84)} = 1.76$	P = 0.145	n.s.
d:IPSC	Vehicle, n = 10 neurons/4 mice; CORT, n = 13 neurons /4 mice	CORT treatment	Two-way ANOVA with repeated measures	$F_{(1,21)} = 0.005$	P = 0.942	n.s.

		Light intensity	Two-way ANOVA with repeated measures	$F_{(4,84)} = 46.38$	$P < 0.001$	$P < 0.001$
		Interaction	Two-way ANOVA with repeated measures	$F_{(4,84)} = 2.829$	$P = 0.030$	$P < 0.05$
e:EPSC	Vehicle, n = 11 neurons/4 mice; CORT, n = 13 neurons/4 mice	CORT treatment	Two-way ANOVA with repeated measures	$F_{(1,22)} = 12.75$	$P = 0.002$	$P < 0.01$
		Light intensity	Two-way ANOVA with repeated measures	$F_{(4,88)} = 68.73$	$P < 0.001$	$P < 0.001$
		Interaction	Two-way ANOVA with repeated measures	$F_{(4,88)} = 3.55$	$P = 0.010$	$P < 0.05$
		Vehicle vs CORT 0.5 μW	Bonferroni post hoc comparison		$P > 0.999$	n.s.
		Vehicle vs CORT 1 μW	Bonferroni post hoc comparison		$P > 0.999$	n.s.
		Vehicle vs CORT 2 μW	Bonferroni post hoc comparison		$P = 0.186$	n.s.
		Vehicle vs CORT 5 μW	Bonferroni post hoc comparison		$P = 0.002$	$P < 0.01$
		Vehicle vs CORT 10 μW	Bonferroni post hoc comparison		$P < 0.001$	$P < 0.001$
e:IPSC	Vehicle, n = 11 neurons/4 mice; CORT, n = 13 neurons/4 mice	CORT treatment	Two-way ANOVA with repeated measures	$F_{(1,22)} = 1.429$	$P = 0.245$	n.s.
		Light intensity	Two-way ANOVA with repeated measures	$F_{(4,88)} = 43.75$	$P < 0.001$	$P < 0.001$
		Interaction	Two-way ANOVA with repeated measures	$F_{(4,88)} = 1.445$	$P = 0.226$	n.s.
f	Same as in (d)	CORT treatment	Two-way ANOVA with repeated measures	$F_{(1,21)}= 0.516$	$P = 0.481$	n.s.
		Light intensity	Two-way ANOVA with repeated	$F_{(4,84)} = 23.26$	$P < 0.001$	$P < 0.001$

			measures			
		Interaction	Two-way ANOVA with repeated measures	$F_{(4,84)}$ = 1.953	P = 0.109	n.s.
g	Same as in (e)	CORT treatment	Two-way ANOVA with repeated measures	$F_{(1,22)}$ = 13.01	P = 0.002	P < 0.01
		Light intensity	Two-way ANOVA with repeated measures	$F_{(4,88)}$ = 21.89	P < 0.001	P < 0.001
		Interaction	Two-way ANOVA with repeated measures	$F_{(4,88)}$ = 3.811	P = 0.007	P < 0.01
		Vehicle vs CORT 0.5 μW	Bonferroni post hoc comparison		P > 0.999	n.s.
		Vehicle vs CORT 1 μW	Bonferroni post hoc comparison		P = 0.124	n.s.
		Vehicle vs CORT 2 μW	Bonferroni post hoc comparison		P = 0.001	P < 0.01
		Vehicle vs CORT 5 μW	Bonferroni post hoc comparison		P = 0.018	P < 0.05
		Vehicle vs CORT 10 μW	Bonferroni post hoc comparison		P = 0.013	P < 0.05
**Supplementary Figure 6**						
c:EPSC	Placebo, n = 11 neurons/4 mice; CORT, n = 12 neurons/4 mice	CORT treatment	Two-way ANOVA with repeated measures	$F_{(1,21)}$ = 0.091	P = 0.766	n.s.
		Light intensity	Two-way ANOVA with repeated measures	$F_{(4,84)}$ = 87.95	P < 0.001	P < 0.001
		Interaction	Two-way ANOVA with repeated measures	$F_{(4,84)}$ = 4.311	P = 0.003	P < 0.01
c:IPSC	Placebo, n = 11 neurons/4 mice; CORT, n = 12 neurons/4 mice	CORT treatment	Two-way ANOVA with repeated measures	$F_{(1,21)}$ = 0.008	P = 0.930	n.s.
		Light intensity	Two-way ANOVA with repeated	$F_{(4,84)}$ = 34.26	P < 0.001	P < 0.001

			measures			
		Interaction	Two-way ANOVA with repeated measures	$F_{(4,84)}$ = 1.854	P = 0.126	n.s.
d:EPSC	Placebo, n = 16 neurons/5 mice; CORT, n = 12 neurons/4 mice	CORT treatment	Two-way ANOVA with repeated measures	$F_{(1,26)}$ = 8.485	P = 0.007	P < 0.01
		Light intensity	Two-way ANOVA with repeated measures	$F_{(4,104)}$ = 79.24	P < 0.001	P < 0.001
		Interaction	Two-way ANOVA with repeated measures	$F_{(4,104)}$ = 9.72	P < 0.001	P < 0.001
		Placebo vs CORT 0.5 µW	Bonferroni post hoc comparison		P > 0.999	n.s.
		Placebo vs CORT 1 µW	Bonferroni post hoc comparison		P = 0.447	n.s.
		Placebo vs CORT 2 µW	Bonferroni post hoc comparison		P = 0.033	P < 0.05
		Placebo vs CORT 5 µW	Bonferroni post hoc comparison		P < 0.001	P < 0.001
		Placebo vs CORT 10 µW	Bonferroni post hoc comparison		P < 0.001	P < 0.001
d:IPSC	Placebo, n = 16 neurons/5 mice; CORT, n = 12 neurons/4 mice	CORT treatment	Two-way ANOVA with repeated measures	$F_{(1,26)}$ = 0.698	P = 0.411	n.s.
		Light intensity	Two-way ANOVA with repeated measures	$F_{(4,104)}$ = 43.43	P < 0.001	P < 0.001
		Interaction	Two-way ANOVA with repeated measures	$F_{(4,104)}$ = 1.505	P =0.206	n.s.
e	Same as in (c)	CORT treatment	Two-way ANOVA with repeated measures	$F_{(1,21)}$ = 0.064	P = 0.803	n.s.
		Light intensity	Two-way ANOVA with repeated measures	$F_{(4,84)}$ = 4.562	P = 0.002	P < 0.01

		Interaction	Two-way ANOVA with repeated measures	$F_{(4,84)} = 0.343$	P =0.848	n.s.
f	Same as in (d)	CORT treatment	Two-way ANOVA with repeated measures	$F_{(1,26)} = 18.03$	P = 0.0002	P < 0.001
		Light intensity	Two-way ANOVA with repeated measures	$F_{(4,104)} = 14.44$	P < 0.001	P < 0.001
		Interaction	Two-way ANOVA with repeated measures	$F_{(4,104)} = 3.132$	P = 0.018	P < 0.05
		Placebo vs CORT 0.5 µW	Bonferroni post hoc comparison		P > 0.999	n.s.
		Placebo vs CORT 1 µW	Bonferroni post hoc comparison		P < 0.001	P < 0.001
		Placebo vs CORT 2 µW	Bonferroni post hoc comparison		P < 0.001	P < 0.001
		Placebo vs CORT 5 µW	Bonferroni post hoc comparison		P = 0.021	P < 0.05
		Placebo vs CORT 10 µW	Bonferroni post hoc comparison		P = 0.060	n.s.
**Supplementary Figure 7**						
b	Control, n = 85 neurons/23 mice; CRS, n = 99 neurons/27 mice	Control vs CRS	Two-tailed unpaired t test		P = 0.100	n.s.
d	Control, n = 96 neurons/23 mice; CRS, n = 99 neurons/27 mice	Control vs CRS	Two-tailed unpaired t test		P < 0.001	P < 0.001
**Supplementary Figure 8**						
b	n = 10 mice		One-way ANOVA with repeated measures	$F_{(2,18)} = 0.358$	P = 0.704	n.s.
c	n = 10 mice		One-way ANOVA with repeated measures	$F_{(2,18)} = 0.237$	P = 0.792	n.s.
e	n = 7 mice		One-way ANOVA with repeated	$F_{(2,12)} = 0.258$	P = 0.776	n.s.

			measures			
f	n = 7 mice		One-way ANOVA with repeated measures	$F_{(2,12)} = 0.452$	P = 0.646	n.s.
h	n = 7 mice		One-way ANOVA with repeated measures	$F_{(2,12)} = 0.258$	P = 0.777	n.s.
i	n = 7 mice		One-way ANOVA with repeated measures	$F_{(2,12)} = 0.168$	P = 0.847	n.s.
**Supplementary Figure 9**						
c	n = 6 mice		Two-tailed paired t test		P = 0.451	n.s.
d	n = 6 mice		Two-tailed paired t test		P = 0.393	n.s.
e	n = 6 mice		Two-tailed paired t test		P = 0.303	n.s.
f	n = 5 mice		Two-tailed paired t test		P = 0.506	n.s.
g	n = 6 mice		Two-tailed paired t test		P = 0.432	n.s.
h	n = 8 mice		Two-tailed paired t test		P = 0.544	n.s.
j	n = 6 mice		Two-tailed paired t test		P = 0.903	n.s.
k	n = 6 mice		Two-tailed paired t test		P = 0.419	n.s.
l	n = 5 mice		Two-tailed paired t test		P = 0.961	n.s.
m	n = 7 mice		Two-tailed paired t test		P = 0.979	n.s.
n	n = 6 mice		Two-tailed paired t test		P = 0.667	n.s.
o	n = 6 mice		Two-tailed paired t test		P = 0.654	n.s.
