# Supplementary material for: Identification of a prefrontal cortex-to-amygdala pathway for chronic stress-induced anxiety
Source: Nat Commun. 2020 May 6;11:2221. doi: 10.1038/s41467-020-15920-7 (PMC7203160; doi:10.1038/s41467-020-15920-7)
Supplement: Supplementary file 3 — Reporting Summary [file 41467_2020_15920_MOESM3_ESM.pdf]

nature research

Corresponding author(s): Bing-Xing Pan

Last updated by author(s): 2020/02/26

# Reporting Summary

Nature Research wishes to improve the reproducibility of the work that we publish. This form provides structure for consistency and transparency in reporting. For further information on Nature Research policies, see Authors & Referees and the Editorial Policy Checklist.

## Statistics

For all statistical analyses, confirm that the following items are present in the figure legend, table legend, main text, or Methods section.

| n/a | Confirmed | |
|---|---|---|
| ☐ | ☒ | The exact sample size (*n*) for each experimental group/condition, given as a discrete number and unit of measurement |
| ☐ | ☒ | A statement on whether measurements were taken from distinct samples or whether the same sample was measured repeatedly |
| ☐ | ☒ | The statistical test(s) used AND whether they are one- or two-sided<br>*Only common tests should be described solely by name; describe more complex techniques in the Methods section.* |
| ☐ | ☒ | A description of all covariates tested |
| ☐ | ☒ | A description of any assumptions or corrections, such as tests of normality and adjustment for multiple comparisons |
| ☐ | ☒ | A full description of the statistical parameters including central tendency (e.g. means) or other basic estimates (e.g. regression coefficient) AND variation (e.g. standard deviation) or associated estimates of uncertainty (e.g. confidence intervals) |
| ☐ | ☒ | For null hypothesis testing, the test statistic (e.g. *F*, *t*, *r*) with confidence intervals, effect sizes, degrees of freedom and *P* value noted<br>*Give P values as exact values whenever suitable.* |
| ☒ | ☐ | For Bayesian analysis, information on the choice of priors and Markov chain Monte Carlo settings |
| ☒ | ☐ | For hierarchical and complex designs, identification of the appropriate level for tests and full reporting of outcomes |
| ☒ | ☐ | Estimates of effect sizes (e.g. Cohen's *d*, Pearson's *r*), indicating how they were calculated |

*Our web collection on statistics for biologists contains articles on many of the points above.*

## Software and code

Policy information about availability of computer code

| | |
|---|---|
| Data collection | Electrophysiological data were collected using HEKA's PatchMaster software (version 2.53). Behavioral data were collected using video-tracking system (Med Associates Inc., Farifax, VT). Confocal immunofluorescence images were taken by using a scanning laser microscope (Olympus FV1000, Tokyo, Japan). |
| Data analysis | Electrophysiological data were analyzed by Origin 8.5. Behavioral data were analyzed by video-tracking system (Med Associates Inc., Farifax, VT). All statistical analysis were performed by GraphPad Prism 7 (Graphpad Software). |

For manuscripts utilizing custom algorithms or software that are central to the research but not yet described in published literature, software must be made available to editors/reviewers. We strongly encourage code deposition in a community repository (e.g. GitHub). See the Nature Research guidelines for submitting code & software for further information.

## Data

Policy information about availability of data

All manuscripts must include a data availability statement. This statement should provide the following information, where applicable:

- Accession codes, unique identifiers, or web links for publicly available datasets
- A list of figures that have associated raw data
- A description of any restrictions on data availability

The data supporting the findings of this study are available within the paper and Supplementary Information files, or from the corresponding author upon reasonable request. A reporting summary for this article is available as a Supplementary Information file. The source data underlying Figs. 1f-h, m-o, t-v, 2f-i, 3b, d, f, h, 4b, d, f, g, i, j, l, m, o, p, 5d-m, 6c, e, h, I, k, l, 7d-f, h-j, l-n, Supplementary Figs. 1c, e, 2d-h, 3d-h, 4b, d, f, h, 5d-g, 6c-f, 7b, d, 8b, c, e, f, h, i, 9c-e, g-i, k-m and o-q are provided as a Source Data file.

# Field-specific reporting

Please select the one below that is the best fit for your research. If you are not sure, read the appropriate sections before making your selection.

☒ Life sciences ☐ Behavioural & social sciences ☐ Ecological, evolutionary & environmental sciences

For a reference copy of the document with all sections, see nature.com/documents/nr-reporting-summary-flat.pdf

# Life sciences study design

All studies must disclose on these points even when the disclosure is negative.

| | |
|---|---|
| Sample size | No statistical methods were used to predetermine the sample sizes, but the sizes were based on our previous studies performing similar experiments (Liu et al, Biol. Psychiatry, 2017, 81: 990-1002, Zhang et al, Biol. Psychiatry, 2019, 85: 189-201.) and convention in the field. |
| Data exclusions | Pre-established exclusion criteria for experimental data points included lack of accurate stereotaxic targeting and/or viral expression based on post-mortem brain tissue analyses. No outliers were excluded in this manuscript. |
| Replication | The data in Figs. 1b, j, q, 2b, 7b and Supplementary Figs. 2b, 3b were repeated for three times and similar results were observed |
| Randomization | Mice injected with retrobeads/viral expression were randomly assigned to control or stress groups. For behavioral experiments, mice were randomly selected to initially receive either the control or stress treatment. |
| Blinding | For the physiological experiments, the investigators were blind to the group assignment during data collection and analysis. The behavior experiments were performed using an automated computer systems, and data were collected and analyzed in an automated and unbiased way, hence no blinding was necessary. |

# Reporting for specific materials, systems and methods

We require information from authors about some types of materials, experimental systems and methods used in many studies. Here, indicate whether each material, system or method listed is relevant to your study. If you are not sure if a list item applies to your research, read the appropriate section before selecting a response.

## Materials & experimental systems

| n/a | Involved in the study | |
|---|---|---|
| ☒ | ☐ | Antibodies |
| ☒ | ☐ | Eukaryotic cell lines |
| ☒ | ☐ | Palaeontology |
| ☐ | ☒ | Animals and other organisms |
| ☒ | ☐ | Human research participants |
| ☒ | ☐ | Clinical data |

## Methods

| n/a | Involved in the study | |
|---|---|---|
| ☒ | ☐ | ChIP-seq |
| ☒ | ☐ | Flow cytometry |
| ☒ | ☐ | MRI-based neuroimaging |

## Animals and other organisms

Policy information about studies involving animals; ARRIVE guidelines recommended for reporting animal research

| | |
|---|---|
| Laboratory animals | Male C57BL/6J mice (5-10 weeks) were used in this study. |
| Wild animals | No wild animals were used in this study. |
| Field-collected samples | No field-collected samples were used in this study. |
| Ethics oversight | All experimental procedures were in accordance with the guidelines of the National Institutes of Health and approved by the Institutional Animal Care and Use Committee of Nanchang University. |

Note that full information on the approval of the study protocol must also be provided in the manuscript.
